# Assessment of ^213^Bi-anti-EGFR MAb treatment efficacy in malignant cancer cells with [1-^13^C]pyruvate and [^18^F]FDG

**DOI:** 10.1038/s41598-019-44484-w

**Published:** 2019-06-05

**Authors:** Benedikt Feuerecker, Michael Michalik, Christian Hundshammer, Markus Schwaiger, Frank Bruchertseifer, Alfred Morgenstern, Christof Seidl

**Affiliations:** 1 Technical University of Munich, School of Medicine, Klinikum rechts der Isar, Department of Nuclear Medicine, Munich, Germany; 2 German Cancer Consortium (DKTK), partner site Munich and German Cancer Research Center (DKFZ), Heidelberg, Germany; 3 Department of Chemistry, Technical University of Munich, Garching, Germany; 4 Munich School of Bioengineering, Technical University of Munich, Garching, Germany; 5 European Commission, Joint Research Centre, Directorate for Nuclear Safety and Security, Karlsruhe, Germany; 6 Technical University of Munich, School of Medicine, Klinikum rechts der Isar, Department of Obstetrics and Gynecology, Munich, Germany

**Keywords:** Cell death, Cancer metabolism

## Abstract

Evaluation of response to therapy is among the key objectives of oncology. A new method to evaluate this response includes magnetic resonance spectroscopy (MRS) with hyperpolarized ^13^C-labelled metabolites, which holds promise to provide new insights in terms of both therapeutic efficacy and tumor cell metabolism. Human EJ28Luc urothelial carcinoma and LN18 glioma cells were treated with lethal activity concentrations of a ^213^Bi-anti-EGFR immunoconjugate. Treatment efficacy was controlled via analysis of DNA double-strand breaks (immunofluorescence γH2AX staining) and clonogenic survival of cells. To investigate changes in metabolism of treated cells vs controls we analyzed conversion of hyperpolarized [1-^13^C]pyruvate to [1-^13^C]lactate via MRS as well as viability of cells, lactate formation and lactate dehydrogenase activity in the cellular supernatants and [^18^F]FDG uptake in treated cells vs controls, respectively. Treatment of malignant cancer cells with ^213^Bi-anti-EGFR-MAb induced intense DNA double-strand breaks, resulting in cell death as monitored via clonogenic survival. Moreover, treatment of EJ28Luc bladder cancer cells resulted in decreased cell viability, [^18^F]FDG-uptake and an increased lactate export. In both EJ28Luc and LN18 carcinoma cells treatment with ^213^Bi-anti-EGFR-MAb triggered a significant increase in lactate/pyruvate ratios, as measured with hyperpolarized [1-^13^C]pyruvate. Treatment with ^213^Bi-anti-EGFR-MAb resulted in an effective induction of cell death in EJ28Luc and LN18 cells. Lactate/pyruvate ratios of hyperpolarized [1-^13^C]pyruvate proved to detect early treatment response effects, holding promise for future clinical applications in early therapy monitoring.

## Introduction

Among various solid cancers, a common feature of cancer cells has been described by Warburg in the 1920’s implying that cancer cells exhibit increased glycolysis even in the presence of oxygen^1^. This remarkable aerobic glycolysis has been exploited in the context of tumor imaging, notably in positron emission tomography (PET), using the glucose analogue 2-deoxy-2-[^18^]fluoro-D-glucose ([^18^F]FDG) labelled with the positon emitter ^18^F^2^. Recently, new methods have proven to unveil new insights into cancer metabolism. As one of them, intracellular pathways can be picked up by using hyperpolarized ^13^C-labeled metabolic probes which monitor fast metabolic pathways in real-time^3^.

Within the last years several research groups have elucidated the potential benefits of applying hyperpolarized ^13^C-labeled metabolic probes in the setting of therapy monitoring in cancerous diseases and as imaging biomarkers for early cancer detection as well as treatment response evaluation^4–13^. The elevated lactate dehydrogenase (LDH) activity in cancer cells satisfies the increased energy demand of proliferating cancer cells by metabolizing pyruvate to lactate even in the presence of oxygen. With the implementation of a new technique called dynamic nuclear polarization, traditional hurdles such as long acquisition times of using ^13^C-labeled compounds for measurements using magnetic resonance spectroscopy (MRS) could be overcome, thus allowing measurements of fast metabolic processes *in vitro* and *in vivo*. LDH exhibits rapid kinetics, therefore representing a fast metabolic intracellular process. For *in vitro* measurements of enzymatic activity, data acquisition is based on the presence of pyruvate and lactate peaks in the observed magnetic resonance spectra, providing the means to calculate the pyruvate to lactate conversion. Compared to the measurement of cellular [^18^F]FDG uptake, which merely monitors [^18^F]FDG incorporation up to the conversion with hexokinase-2, measurement of pyruvate to lactate conversion allows an insight of metabolic processes further downstream in the glycolytic signal transduction pathways.

As demonstrated in a large number of studies, the alpha-emitter ^213^Bi coupled to various targeting compounds effectively eradicated tumor cells *in vitro* and *in vivo* due to its high linear energy transfer^14,15^. Overexpression of the epidermal growth factor receptor (EGFR) has been documented in several malignancies including bladder cancer and glioma^14,16^. In an animal model of human bladder cancer ^213^Bi-anti-EGFR immunoconjugates showed effective eradication of human EJ28Luc tumor cells and therefore significantly prolonged overall survival of the treated animals^14,17^. Meanwhile, ^213^Bi-anti-EGFR-MAb has also been administered successfully in a pilot study encompassing 12 patients suffering from bladder cancer^18^. Moreover, the alpha-emitters ^213^Bi and ^211^At have demonstrated therapeutic efficacy in glioma both targeting the neurokinin type 1 receptor^19,20^ and extracellular tenascin^21^.

To date, several studies have evaluated alterations in gene expression following targeted treatment with alpha-emitters^22–24^. Changes in cellular metabolism induced by alpha-emitters could be investigated via the uptake and cellular accumulation of [^18^F]FDG. Once internalized, phosphorylation of the glucose analog [^18^F]FDG prevents its release from the cell. However, phosphorylated [^18^F]FDG is not metabolized via glycolysis due to the lack of the 2-hydroxyl group^2^. Other techniques that can be used for *in vivo* monitoring of metabolism employ hyperpolarization of molecules that contain ^13^C.

After the injection of a specific solution containing a ^13^C hyperpolarized compound, metabolic changes can be monitored immediately as a result of conversion of the probe. For example, detection of conversion of hyperpolarized [1-^13^C]pyruvate to [1-^13^C]lactate can be accomplished via magnetic resonance imaging (MRI) thus visualizing metabolic pathways noninvasively that are involved in cellular reactions to external damaging agents^25^. Therefore, observed changes in the elevated lactate turnover (characteristic of tumor cells: Warburg effect) could be indicative of the damaging power of an administered compound.

In the present study we focused on the assessment of the treatment response of EJ28Luc bladder cancer and LN18 glioma cells with hyperpolarized [1-^13^C]pyruvate. For this purpose, we used magnetic resonance spectroscopy (MRS) to assess the treatment effects of ^213^Bi-anti-EGFR-MAb by calculation of the conversion of pyruvate to lactate, via spectroscopy of hyperpolarized [1-^13^C]pyruvate. To further investigate metabolic alterations upon treatment, we monitored [^18^F]FDG-uptake into treated and control cells. Efficacy of treatment with ^213^Bi-anti-EGFR-MAb was monitored via clonogenic survival of cells and detection of cellular DNA double-strand breaks.

## Materials and Methods

### Cell lines

The human urothelial carcinoma cell line EJ28Luc, isolated from a primary bladder carcinoma was grown in RPMI medium supplemented with 10% fetal calf serum and 1% nonessential amino acids (Biochrom, Berlin, Germany) in a humified atmosphere containing 5% CO_2_. Transfection of cells was previously carried out with the plasmid pcDNA3.1 containing the coding sequence of firefly (Photinus pyralis) luciferase^14^. The human glioma cell line LN18 was cultured in RPMI medium supplemented with 10% fetal calf serum at 5% CO_2_. Cells were harvested with Trypsin/EDTA (0.05%/0.02%; Biochrom).

### Coupling of ^213^Bi to anti-EGFR-MAb

Anti-EGFR-MAb (cetuximab; Merck, Darmstadt, Germany) was conjugated with the ^213^Bi chelating compound SCN-CHX-A“-diethylenetriaminepentaacetic acid (DTPA) (Macrocyclics, Plano, USA) as previously described^26^. The α-emitter ^213^Bi was eluted from an ^225^Ac/^213^Bi generator system provided by the Directorate for Nuclear Safety and Security, JRC, EC, Karlsruhe^27,28^. CHX-A“-DTPA-chelated anti-EGFR-MAb (100 µg) was incubated with the ^213^Bi eluate (37–148 MBq) in 0.4 M ammonium acetate buffer at pH 5.3 for 7 min at room temperature. Unbound ^213^Bi was separated via size-exclusion chromatography. Purity of ^213^Bi-anti-EGFR conjugates was controlled via instant thin-layer chromatography as described earlier^29^.

### Determination of ^213^Bi-anti-EGFR-mAb binding to the analyzed cells

For evaluation of cell binding of the alpha-emitter radioimmunoconjugate, cells (LN18, EJ28Luc, OVCAR-3, 3 × 10^6^ each in 0.5 mL cell culture medium) were incubated with ^213^Bi-anti-EGFR-mAb (37 kBq, approximately 50 ng) for 30 min on ice. Subsequently, cells were re-suspended with 0.5 ml PBS each and centrifuged (1,200 rpm, 3 min). The resulting supernatant 1 was aspirated and the cells of the pellet were re-suspended with 0.5 ml PBS. Following another centrifugation and aspiration of supernatant 2, ^213^Bi activity of the cellular pellet as well as of supernatants 1 and 2 was quantified using a γ-counter (1480 Wizard TM3; Wallac). The activity measured in the cellular pellet relative to the total activity (pellet + supernatant 1 + supernatant 2) represents the percentage of bound ^213^Bi-anti-EGFR conjugates.

### Assessment of cell viability/proliferation

Cell viability/proliferation was evaluated at different time points after incubation of LN18 and EJ28Luc cells with ^213^Bi-anti-EGFR-MAb via microscopical observation. For this purpose approx. 5 × 10^4^ cells were seeded per culture flask (25 cm^2^) and were left to adhere overnight at 37 °C and 5% CO_2_. On the following day, cells were incubated for 3 h with 1.48 MBq/ml of ^213^Bi-anti-EGFR-MAb or mock-treated (equivalent volume of PBS). Subsequently medium was removed, cells were washed once with PBS and fresh cell culture medium was added. Cells were incubated at 37 °C/5% CO_2_ and viability/proliferation was monitored microscopically at numerous time points between 48 h (2 d) and 768 h (32 d) after treatment.

### Immunofluorescence detection of DNA double-strand breaks (DSB) via γH2AX after ^213^Bi-anti-EGFR-MAb treatment of cells

LN18 and EJ28Luc cells were seeded in 2-well chamber slides (Thermo Fisher Scientific, Munich, Germany) at approx. 2.5 × 10^4^ cells/well and incubated overnight for adhesion. Cells were treated with ^213^Bi-anti-EGFR-MAb (1.48 MBq/ml) in one mL of culture medium for 3 h. Subsequently, medium was aspirated, cells were washed twice with PBS (2 mL) and fixed with 4% formalin for 15 min. Formalin was then removed, cells were washed three times with PBS and permeabilized with methanol (−20 °C, 5 min). For immunostaining cells were initially blocked with 5% FCS in PBS for 30 min. The primary antibody (anti-γH2AX, Millipore, Germany) was diluted 1:200 in PBS and applied for 90 min at RT. Subsequently cells were washed three times for 10 min with PBS + 1% FCS. Cells were incubated with the secondary antibody (goat anti-mouse IgG FITC conjugate, 1 mg/ml, 1:200, Millipore, Germany) for 1 h at RT. Right before dismantling of the chamber from the slide, cells were washed four times (10 min) in PBS. Images were acquired with a fluorescence microscope (Keyence, Neu-Isenburg, Germany) after embedding of cells in DAPI-containing mounting medium (Prolong® Gold Antifade Reagent, Thermo Fisher Scientific, Munich Germany).

### Determination of extracellular lactate concentration

Extracellular lactate concentration in the cell culture medium was measured 48 h after treatment with ^213^Bi-anti-EGFR-MAb, using a commercial lactate assay kit (Roche, Penzberg, Germany). For this purpose, cells were seeded as monolayers in 96 well plates (2.5 × 10^4^ cells/well) and allowed to adhere overnight. ^213^Bi-anti-EGFR-MAb (1.48 MBq/ml) or PBS (mock-treatment) was added and cells were incubated for 3 h at 37 °C. Thereafter, medium was exchanged and cells were incubated for another 48 h. The cell culture medium was collected and stored at −80 °C until further processing. Lactate concentration measurements were carried out according to the manufacturers’ instructions. A standard curve using known lactate concentrations was prepared for every experiment (n = 3). The assay is based on the enzymatic conversion of pyruvate to lactate that is visualized via a colorimetric change. Concentrations of lactate were noted as nmol/µl.

### Measurement of lactate dehydrogenase activity

Lactate dehydrogenase (LDH) activity in the cell culture medium was measured 48 h after treatment with ^213^Bi-anti-EGFR-MAb, using a commercial LDH assay kit (Sigma Aldrich, Taufkirchen, Germany). For this purpose, a part of the medium that was previously collected for determination of the extracellular lactate concentration and stored at −80 °C was used. The medium was gently thawed and samples were processed according to the manufacturers instructions. A standard curve using known amounts of LDH was prepared. The assay is based on the enzymatic reduction of nicotinamide adenine dinucleotide (NAD) to NADH by LDH that is visualized via a colorimetric change. LDH activity was noted as mU/ml.

### [^18^F]FDG-uptake after exposure of cancer cells to ^213^Bi-anti-EGFR-MAb

In order to evaluate the effects of ^213^Bi-anti-EGFR-MAb treatment on glucose metabolism of LN18 and EJ28Luc cells, [^18^F]FDG-uptake was quantified. Approx. 1 × 10^5^ cells were seeded per well (24-well plates, Greiner Bio-One, Germany) and allowed to adhere overnight. The next day, cells were incubated with 1.48 MBq/ml ^213^Bi-anti-EGFR-MAb for 3 h or mock-treated with an equivalent volume of PBS. Following exchange of cell culture medium, cells were incubated for 48 h at 37 °C and 5% CO_2_. Subsequently cells were washed twice with glucose-free medium (DMEM) and 0.185 MBq/ml of [^18^F]FDG was added per well to the cells in glucose-free medium. Cells were incubated for 60 min at 37 °C and 5% CO_2_ and then put on ice, in order to stop [^18^F]FDG uptake. To remove unincorporated [^18^F]FDG, cells were washed twice with ice-cold PBS and the cell pellet was finally lysed with 1 M NaOH. ^18^F-activity representing [^18^F]FDG uptake was measured in a gamma counter (Wizzard^2^, Perkin Elmer, Germany). Experiments were carried out in triplicates.

### Conversion of hyperpolarized [1-^13^C]pyruvate after incubation of cancer cells with ^213^Bi-anti-EGFR-MAb

For measurements of metabolic conversion of [1-^13^C]pyruate to [1-^13^C]lactate, a 20 mM pyruvate solution doped with 15 mM OX063 trityl radical (Oxford instruments, Abingdon, Oxfordshire, UK) was hyperpolarized using a HyperSense dynamic nuclear polarizer (Oxford Instruments) for approximately 45 min. Hyperpolarization was carried out at a magnetic field of ~3.35 T, temperature of ~1.4 K, microwave frequency of ~94.1 GHz and microwave power of ~100 mW. The hyperpolarized sample was rapidly dissolved with ~4 mL of a dissolution buffer (20 mM NaOH, 20 mM Tris, physiological pH) at ~185 °C (pressure, ~10 bar) and 250 µl of this solution (20 mM of hyperpolarized pyruvate) were mixed with the cells (cells suspended in 750 µl cell culture medium), previously transferred in a spectrometer compatible 5 mm nuclear magnetic resonance (NMR) tube^30^. Approximately 2–3 × 10^7^ cells were transferred to the NMR tube immediately before the measurement without the usage of a perfusion system. In the current study, we used an established protocol for acquisition of the carbon magnetic resonance spectra (repetition time (TR) of 3 s and a flip angle of 10°, Spinsolve Carbon, Magritek, Aachen, Germany) demonstrating conversion of pyruvate to lactate. The ^13^C-data were processed using MestReNova software (Mestrelab Research, Santiago de Compostella, Spain). Pyruvate to lactate conversion rates (k_pl_) and LDH activity was calculated using the model-free approach previously presented by Hill *et al*.^31^ and according to the work of Day *et al*.^32^.

The cells used for these experiments were pretreated as follows: EJ28Luc cells and LN18 cells were seeded in culture flasks (approx. 5 × 10^6^ cells per 175 cm^2^ flask). The next day, four culture flaks of EJ28Luc and LN18 cells, respectively, containing approximately 1 × 10^7^ cells each, were incubated with ^213^Bi-anti-EGFR-MAb (1.48 MBq/ml, in a total volume 10 ml) for 3 h. Controls were incubated with equal volumes of PBS. Forty-eight h after treatment cells were detached, transferred to a 5 mm NMR tube and immediately subjected to the spectrometric measurements with regard to conversion of pyruvate to lactate.

### Data analysis and statistical analysis

Quantification of pyruvate to lactate conversion from the hyperpolarization experiments was performed in MatLab (Math Works, Natick, USA). Statistical analysis was carried out using GraphPad Prism (GraphPad inc. Version 6.0) and results were regarded as statistically significant in case of p < 0.05 following a Students’s t-test and Welch’s correction. Data is expressed as mean ± SD.

All methods were carried out in accordance with relevant guidelines and regulations.

## Results

### Binding of ^213^Bi-anti-EGFR-MAb to tumor cells

**Figure 1:**
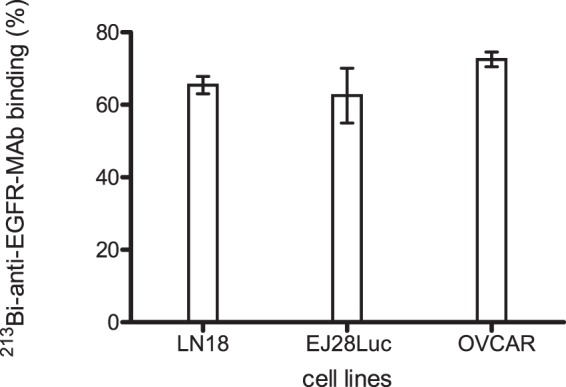
Binding of ^213^Bi-anti-EGFR-MAb to selected tumor cell lines. Binding of ^213^Bi-anti-EGFR-MAb to the cell lines used in this study, LN18 and EJ28Luc, and to the reference cell line OVCAR-3 (3 × 10^6^ cells each) was assayed in triplicate for 30 min on ice to exclude internalization (see Mat. + Meth.). Displayed are mean ± SD.

The proportion of bound antibody compared to unbound substrate as assessed by a binding assay revealed that ^213^Bi-anti-EGFR-MAb showed good binding to LN18 and EJ28Luc cells, 65% ± 2.4% and 63% ± 7.6%, respectively (Fig. 1). The reference cell line OVCAR, known to highly express EGFR on the cell surface, showed 72% binding of ^213^Bi-anti-EGFR-MAb. Therefore, the cell lines investigated in this study, LN18 and EJ28Luc, are well suited for the analysis of effects induced by targeted ^213^Bi-anti-EGFR-MAb treatment.

### Viability/proliferation of EJ28Luc and LN18 cells after treatment with ^213^Bi-anti-EGFR-MAb

Treatment of both EJ28Luc bladder cancer and LN18 glioblastoma cells with ^213^Bi-anti-EGFR-MAb (1.48 MBq/ml) effectively eradicated tumor cells, as monitored via microscopical observation. 48 h to 72 h after treatment, cells were swollen and/or showed distinct features of massive irradiation damage compared to untreated controls. Proliferation could not be observed. Though cells were incubated with lethal activity concentrations of ^213^Bi-anti-EGFR-MAb, disintegration of cells could not be observed before 72 h after initiation of radiation exposure. Cell death was usually completed 96 h after start of radiation exposure. Treatment of EGFR expressing cells with ^213^Bi-anti-EGFR immunoconjugates (1.48 MBq/ml) proved to be lethal for the vast majority of EJ28Luc and LN18 cells. Ten days after treatment, we observed that from 50,000 cells that had been initially seeded, approximately 20 in case of EJ28Luc and 10 in case of LN18 had survived, as deduced from microscopically detectable small nests of proliferating cells. Therefore, ^213^Bi-anti-EGFR-MAb treatment had resulted in eradication of 99.98% (EJ28Luc) and 99.99% (LN18) of tumor cells, respectively.

### Detection of DNA double-strand breaks via γH2AX immunofluorescence after ^213^Bi-anti-EGFR-MAb treatment

**Figure 2:**
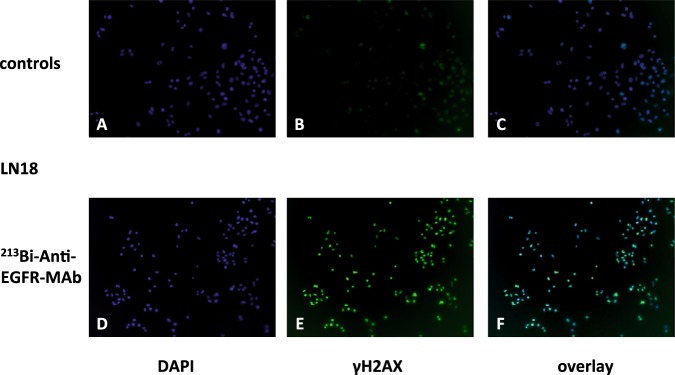
Detection of DNA double-strand breaks via γH2AX staining in LN18 cells after treatment with ^213^Bi-anti-EGFR-MAb. Cells were incubated with ^213^Bi-anti-EGFR-MAb for 3 h at 4 °C to inhibit DNA-repair or mock-treated with PBS (controls). Subsequently cell culture medium was exchanged and cells were incubated for 1 h at 37 °C to initiate DNA-repair. Shortly thereafter, cells were fixed, stained for γH2AX (**B**,**E**) and counterstained with DAPI for visualization of the nuclei (**A**,**D**). Intranuclear foci indicate DNA double-strand breaks induced by the alpha-emitter ^213^Bi (**C**,**F** overlay, representative images).

Upon staining of γH2AX reflecting DNA double-strand breaks, nuclei of LN18 glioblastoma cells showed strong signal enhancements 1 h after treatment with ^213^Bi-anti-EGFR-MAb compared with untreated controls (Fig. 2). Treatment of EJ28Luc bladder carcinoma cells with ^213^Bi-anti-EGFR-MAb caused similar effects as to induction of DNA double-stand breaks (data not shown).

### Determination of the extracellular lactate concentration after ^213^Bi-anti-EGFR-MAb treatment

**Figure 3:**
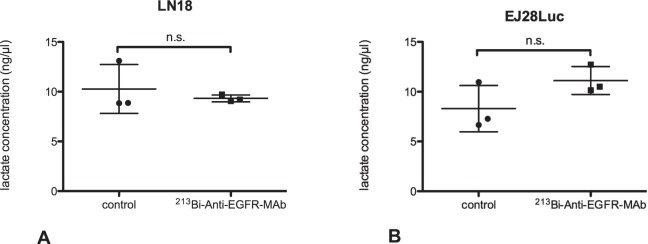
Lactate concentrations in cell culture media of LN18 (**A**) and EJ28Luc (**B**) cells 48 h after ^213^Bi-anti-EGFR-MAb treatment. Lactate concentrations, as determined with a commercial assay kit, did not significantly differ in untreated controls and ^213^Bi-anti-EGFR-MAb treated cells, both in EJ28Luc and LN18 cells. ^213^Bi-anti-EGFR-MAb treatment induced a slightly elevated lactate concentration in EJ28Luc cells and a negligible decrease of lactate concentration in LN18 cells. Displayed are mean ± SD.

As displayed in Fig. 3 the lactate concentration in cell culture medium of EJ28Luc cells treated with ^213^Bi-anti-EGFR-MAb (11.1 ± 0.8 ng/μl) was slightly increased compared to untreated controls (8.3 ± 1.3 ng/ml) (p = 0.14). However, in LN18 cells ^213^Bi-anti-EGFR-MAb treatment induced a slight decrease (p = 0.54): analysis of cell culture medium revealed lactate concentrations of 10.3 ± 1.4 ng/ml and 9.3 ± 0.2 134 ng/ml for controls and ^213^Bi-anti-EGFR-MAb treated cells, respectively.

### Determination of LDH activity after ^213^Bi-anti-EGFR-MAb treatment

**Figure 4:**
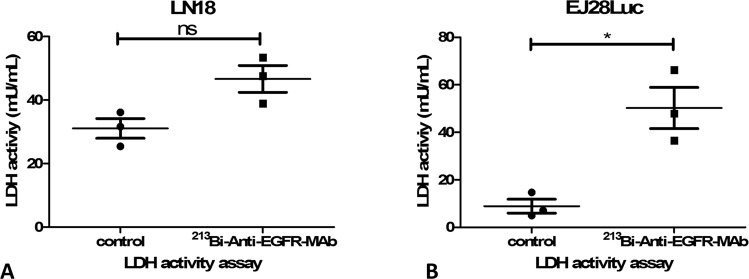
Lactate dehydrogenase activity (LDH) in cell culture media of LN18 (**A**) and EJ28Luc (**B**) cells 48 h after ^213^Bi-anti-EGFR-MAb treatment. LDH, as determined with a commercial assay kit, did significantly differ in untreated controls and ^213^Bi-anti-EGFR-MAb treated cells, in EJ28Luc but not in LN18 cells. ^213^Bi-anti-EGFR-MAb treatment induced an elevated LDH activity in both cell lines indicative of cell death as LDH normally is stored intracellular but not in the extracellular compartment. Displayed are mean ± SD.

As displayed in Fig. 4 the LDH activity in cell culture medium of EJ28Luc cells treated with ^213^Bi-anti-EGFR-MAb (50.2 ± 15.0 mU/ml) was significantly increased compared to untreated controls (8.9 ± 5,1 mU/ml, p = 0.0458). In LN18 cells ^213^Bi-anti-EGFR-MAb treatment induced also an increase, which showed a trend towards statistical significance (p = 0.0591): analysis of cell culture medium revealed a LDH activity of 31.1 ± 5.4 ng/ml and 46.6 ± 7,3 ng/ml for controls and ^213^Bi-anti-EGFR-MAb treated cells, respectively.

### [^18^F]FDG-Uptake after exposure to ^213^Bi-anti-EGFR-MAb

**Figure 5:**
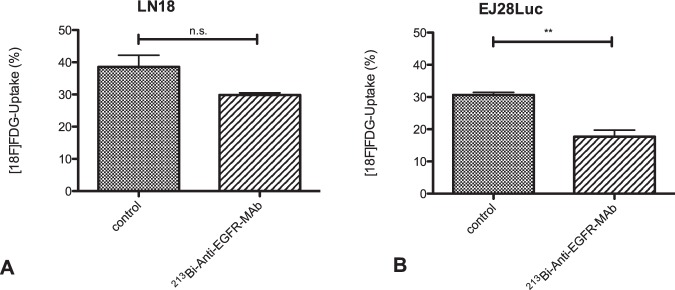
[^18^F]FDG-Uptake in LN18 (**A**) and EJ28Luc (**B**) cells treated with ^213^Bi-anti-EGFR-MAb. The diagrams show the uptake following a 90 min incubation with [^18^F]FDG of cells (three replicates) treated with ^213^Bi-anti-EGFR-MAb or mock-treated with PBS (control). In both cell lines ^213^Bi-anti-EGFR-MAb induced a decrease in [^18^F]FDG uptake. The decrease was statistically significant in EJ28 cells (p = 0.0095) and showed a trend towards statistical significance in LN18 cells (p = 0.054). Displayed are mean ± SD.

Following treatment with ^213^Bi-anti-EGFR-MAb (3 h) and subsequent incubation for 48 h, LN18 cells showed a reduced uptake of [^18^F]FDG after 60 min (p = 0.054) compared with untreated controls (Fig. 5A). Likewise, ^213^Bi-anti-EGFR-MAb treatment of EJ28Luc induced a statistically significant decrease in [^18^F]FDG-uptake (p = 0.0095; Fig. 5B).

### Metabolic conversion of hyperpolarized [1-^13^C]pyruvate after ^213^Bi-anti-EGFR-MAb treatment

Detection of hyperpolarized ^13^C-labelled metabolites in LN18 and EJ28Luc cells resulted in small amounts of lactate and pyruvate-hydrate as well as comparatively large amounts of non-metabolized pyruvate in the spectra of untreated controls and ^213^Bi-anti-EGFR-MAb treated cells.

**Figure 6:**
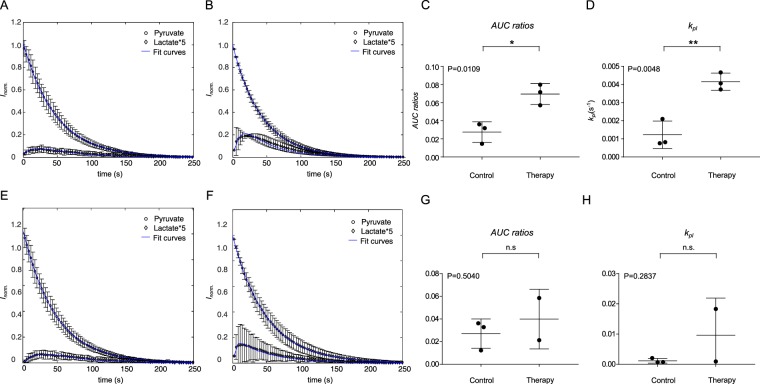
Lactate/pyruvate time intensity curves, (EJ 28Luc: **A** controls, **B** treated; LN18: **E** controls, **F** treated), AUC ratios (**C**: EJ28Luc, **G**: LN18) and k_pl_ values (**D**: EJ28Luc, **H**: LN18) after treatment with ^213^Bi-anti-EGFR-MAb via detection of hyperpolarized [1-^13^C]-labeled compounds. For determination of cellular lactate/pyruvate ratios, LN18 and EJ28Luc cells were incubated either with PBS (control) or ^213^Bi-anti-EGFR-MAb, harvested after 48 h and transferred to NMR tubes. After polarization of [1-^13^C]pyruvate in a DNP polarizer for 35 min, the hyperpolarized [1-^13^C]pyruvate was rapidly injected into the NMR tube containing the cells and transferred into a magnetic resonance spectrometer for detection of pyruvate to lactate conversion. Corresponding sample time intensity curves are shown for EJ28Luc (**A**,**B**) and LN18 (**E**,**F**) cells, indicating an increase of lactate after treatment with ^213^Bi-anti-EGFR-MAb. Incubation with ^213^Bi-anti-EGFR-MAb increased lactate/pyruvate ratios in both cell lines compared to PBS-treated controls (**C** EJ28Luc, **G** LN18). ^213^Bi-anti-EGFR-MAb treatment primarily triggered an increase in the lactate formation in both cell lines that resulted in an increase of the k_pl_ values as indicated in **D** and **H**. Displayed are mean ± SD.

In contrast, amounts of lactate were increased in ^213^Bi-anti-EGFR-MAb treated cells compared to untreated controls, which was also seen in the time intensity curves (Fig. 6A EJ28Luc untreated, 6E LN18 untreated, 6B EJ28Luc and 6F LN18 treated with ^213^Bi-anti-EGFR-Mab, respectively). Therefore, the lactate/pyruvate ratio was higher in cells that were treated with ^213^Bi-anti-EGFR-MAb compared with untreated cells, both in EJ28Luc and LN18 cells (Fig. 6C, 6G). Moreover, increase was statistically significant in EJ28Luc cells (EJ28Luc, p = 0.00244, both n = 3). Untreated EJ28Luc cells showed a lactate/pyruvate ratio of 0.028 ± 0.006 (n = 3). Treatment with ^213^Bi-anti-EGFR-MAb increased the lactate/pyruvate ratio to 0.074 ± 0.009 (n = 3). In the glioblastoma cell line LN18 we observed a lactate/pyruvate ratio of 0.027 ± 0.007 in untreated cells (n = 3). In those cells that were treated with ^213^Bi-anti-EGFR-MAb the lactate/pyruvate ratio increased to 0.048 ± 0.028 (n = 2), representing a statistically non significant increase (p = 0.5850) compared with untreated cells. In some samples we also detected pyruvate hydrate^33^. There was a significant increase of the k_pl_ value in cells treated with ^213^Bi-anti-EGFR-MAb in EJ28Luc cells compared to untreated cells (p = 0.0048; Fig. 6D). In LN18 cells there was also a trend of cells treated with ^213^Bi-anti-EGFR-MAb towards an increase of k_pl_, but this was not statistically significant (p = 0.2837; Fig. 6H).

## Discussion

Monitoring the response to therapeutic treatments remains a challenging question in clinical oncology. To date molecular imaging has proven its capacities in helping to understand treatment associated effects and guiding clinical decisions to sustain, pause or stop scheduled therapies. Among the most important imaging modalities morphological (CT/MRI) and functional (PET) imaging have evolved to provide evidence concerning efficacy of a therapeutic option. With the introduction and broad application of new drugs such as anti-angiogenetic drugs (e.g. bevacizumab) new challenges arise in the fact that these drugs do not cause therapy related reductions of tumor size within a short time frame. But also for other new promising therapeutic options such as targeted treatment with alpha-emitters^34,35^ detection of early response to treatment remains challenging as therapeutic effects also do not occur within the first hours post application^22^. New metabolic tracers might provide detailed information in detection of early treatment response because the standard criteria in morphological imaging defined by the response evaluation criteria in solid tumors (RECIST) cannot uncritically be employed. Such new tracers hold promise to detect subtle changes before morphological changes occur. For example, widely used tracers such as [^18^F]FDG but also new techniques such as hyperpolarized [1-^13^C]pyruvate NMR spectroscopy might provide new insights as to early treatment responses.

In this study we investigated the detection of metabolites of hyperpolarized [1-^13^C]pyruvate with regard to monitoring treatment response using ^213^Bi-anti-EGFR-MAb (the alpha-emitter ^213^Bi coupled to an anti-EGFR antibody) in an *in vitro* system using LN18 and EJ28Luc tumor cells. Beyond, treatment efficacy was evaluated analysing cellular [^18^F]FDG-uptake. Coupling of the alpha-emitter ^213^Bi to the anti-EGFR antibody was successfully accomplished and efficient binding of the radioimmunoconjugate to the analysed cell lines was demonstrated. Following incubation of the cells with ^213^Bi-anti-EGFR-MAb, we examined the efficacy of cellular damage and underlying metabolic alterations.

We demonstrated that evaluation of early response in cells (after 48 h) to treatment is possible. As shown by γH2AX fluorescence staining, induction of DNA double-strand breaks occurred upon treatment with the ^213^Bi-anti-EGFR conjugate. Our results also confirmed effective killing of tumor cells upon treatment with the ^213^Bi-immunoconjugate as shown in previous studies^34,36,37^. We could detect treatment effects, i.e. induction of DNA double-strand breaks that finally resulted in cell death, already 1 h after incubation of cells with ^213^Bi-anti-EGFR-MAb, at a time point where cells did not show any morphological indication of the upcoming cell death. Cell death usually occurs 3–4 days after treatment with lethal activity concentrations of ^213^Bi-immunoconjugates (1.48 MBq/ml). This is due to the cellular rescue program that initiates cell death not before several rounds of DNA repair have failed to restore functionality of the cellular framework^22^.

Furthermore, we investigated the molecular effects of ^213^Bi-anti-EGFR-MAb treatment in LN18 and EJ28Luc tumor cells with two techniques. The first of these techniques -intracellular uptake of [^18^F]FDG - is already in clinical use, the second one - NMR spectroscopy of hyperpolarized [1-^13^C]pyruvate - will be probably translated to the clinic in the future. First-in-human hyperpolarized pyruvate studies of brain metabolism in cases of glial neoplasms and metastases proved to be feasible^38^. Here, NMR of hyperpolarized [1-^13^C]pyruvate has proven to be appropriate for monitoring the early response of both carcinoma cell lines analysed to treatment with ^213^Bi-anti-EGFR-MAb. Using hyperpolarized [1-^13^C]pyruvate, we were able to show, for the first time, that treatment with ^213^Bi-anti-EGFR-MAb resulted in alterations of the lactate/pyruvate ratio *in vitro*, thus reflecting severe cell damage in combination with an obviously up-regulated lactate-dehydrogenase (LDH) activity.

The NMR measurements of pyruvate to lactate conversion using [1-^13^C]pyruvate, as performed 48 h after ^213^Bi-anti-EGFR-MAb treatment, were done using a cellular pellet (see Material and Methods), i.e. without culture medium. Therefore, we could show that 48 h after ^213^Bi-anti-EGFR-MAb treatment, there was an increase in cytosolic LDH activity (see Fig. 6). Moreover, we have detected an increased LDH activity also in cell culture media of ^213^Bi-anti-EGFR-MAb treated cells (see Fig. 4) indicative of an enhanced LDH release from the cells, which could be either due to membrane damage or an increase of the active transport of LDH activity across the cell membrane. Though we have hints that the cell membrane is not damaged as early as 48 h after treatment of cells with ^213^Bi-anti-EGFR-MAb (as deduced from microscopic observations)^22^, a current review on LDH in cancer cells strongly supports the hypothesis that the extracellular increase of LDH is most likely due to membrane leakage, induced by cytotoxic compounds^39^. Moreover, we actually have no evidence that LDH is actively transported into the cell culture medium. Other studies have shown that kinetic values obtained with hyperpolarized magnetic resonance spectroscopy resemble *in vivo* LDH rate constants^3,31,40–42^. As demonstrated previously, LDH mutually converts pyruvate and lactate within the intracellular compartment. A large pool of lactate is often observed in viable tumors compared to pyruvate, allowing for metabolic assessment of a cells’ vitality^43–45^. Increased lactate formation following treatment of cells with ^213^Bi-anti-EGFR-MAb therefore is reflective of an increase of LDH activity compared to untreated controls. Treatment with ^213^Bi-anti-EGFR-MAb caused an increase in the lactate/pyruvate ratio of 37% and 55% in EJ28Luc cells LN18 cells, respectively. Therefore, this technique using hyperpolarized [1-^13^C]pyruvate holds promise to offer new insights as to detection of early metabolic changes upon cancer treatment in clinical oncology. Further studies need to be performed to confirm the results of these *in vitro* analyses also *in vivo*. Moreover, other studies using [1-^13^C]pyruvate have demonstrated that this new technique is a promising option for evaluation of early treatment responses^43,46,47^. As our study focused on applying this technique in an *in vitro* setting, more challenging questions of applying this technique *in vivo* will be addressed in separate studies.

To validate our findings concerning upregulation of LDH activity and elevated lactate/pyruvate ratios as observed via NMR spectroscopy using hyperpolarized [1-^13^C]pyruvate we assayed the lactate content in cell culture media (cell supernatants) of cells after ^213^Bi-anti-EGFR-MAb treatment. In both LN18 and EJ28luc cell lines no significant treatment induced alterations in the lactate concentrations were detected. These findings of the conventional assay might be due to a decreased stability of lactate in the cell culture medium. Further in depth analyses will be needed to clarify these findings.

The tracer [^18^F]FDG has been used in clinical oncology for many decades for determination of responses to treatment and in staging of cancerous diseases. Numerous studies have shown that this technique involving [^18^F]FDG can be easily employed in daily patient care. We used [^18^F]FDG in this study as a “gold-standard” in order to decipher possible effects upon treatment. Our measurements show, that treatment with ^213^Bi-anti-EGFR-MAb resulted in a significant decrease of [^18^F]FDG uptake in EJ28Luc bladder cancer cells, sustaining the hypothesis that alpha radiation results in metabolic alterations. However, in the LN18 cells no significant alteration in [^18^F]FDG uptake was observed after ^213^Bi-anti-EGFR-MAb treatment. Nevertheless, a clear trend towards a reduction of [^18^F]FDG uptake was registered, constituting approx. 23%. We cannot rule out that this is a statistical effect, which causes this data to be not significant. In the EJ28Luc cell line, the reduction of [^18^F]FDG uptake was almost 42%. Beyond, it is possible, that the LN18 glioblastoma cell line is less dependent on glucose compared to the EJ28Luc bladder carcinoma cell line. Most likely the effects measured in our study are completely based on the induction of cell death. Therefore, possible intracellular metabolic adaptions triggering a metabolic switch that might result in incorporation of metabolites such as glutamine or fatty acids instead of glucose seem less likely to explain our findings. It was shown previously, that glucose deprivation led to an increase of lactate dehydrogenase A (LDHA) and isocitrate dehydrogenase 1 (IDH1) in LN18 cells, which demonstrates the adaptive potential of these cells upon cellular stress^48^. Moreover, in preparation of cell death ATP becomes increasingly depleted. Therefore, activity of ATP-dependent hexokinase involved in phosphorylation of [^18^F]FDG could decrease finally resulting in an reduced trapping of [^18^F]FDG in the intracellular compartment.

Notably, the differential information that can be gained by applying both hyperpolarized [1-^13^C]pyruvate and [^18^F]FDG might hold promise to allow for better understanding of tumor biology and also phenotyping tumor cells and tissues^49–52^. It was reported that the relation of both lactate generation and [^18^F]FDG-uptake in cancers relates on cancer type^49–52^. In this study, we noticed, that treatment with ^213^Bi-anti-EGFR-MAb resulted in reduced uptake of [^18^F]FDG but increased area under the curve (AUC) ratios as assessed through hyperpolarized [1-^13^C]pyruvate. It was previously reported, that lactate generation and [^18^F]FDG-uptake can be related, a finding that is in agreement with the Warburg effect, but their relation depends on the type of cancer tissue^52^. In our study, we measured a mismatch with reduced [^18^F]FDG-uptake and increased hyperpolarized lactate production upon treatment. Reduction of [^18^F]FDG-uptake as shown in our study following incubation of tumor cells with ^213^Bi-anti-EGFR-MAb, was also shown previously as a result of cytotoxic therapy^13,53^. In line with a preceding study, which focused on evaluation of treatment response of *in vivo* tumors to irradiation, we also noticed a significant increase of hyperpolarized lactate formation and k_pl_^54^. Therefore, the information of both measurements is additive as one would assume a decreased lactate formation based on the [^18^F]FDG-uptake measurements. Hence, combining both techniques enhances understanding tumor response to treatment in a preclinical setup. Clearly, this can only be a first step in order to understand treatment related effects of antitumor substances. A detailed understanding of the underlying effects might help when translating these techniques to preclinical and clinical applications, e.g. in the context of measuring treatment effects in bladder cancer^18^. Preclinical studies focusing on validation of our findings *in vivo* will further increase our understanding of the effects of ^213^Bi-anti-EGFR-MAb treatment and allow to draw solid conclusions. Combining both magnetic resonance spectroscopy and PET imaging has already been successfully translated onto small animal and also clinical PET/MRI scanners, further enhancing the potential to gain valuable insights into tumor metabolism *in vivo* through a multimodal (imaging) approach^55–57^.

In summary, our study shows that assessment of early treatment effects in cancer cells using hyperpolarized [1-^13^C]pyruvate provides insights of early treatment related effects in LN18 and EJ28luc cancer cells exposed to ^213^Bi-immunoconjugates. Follow-up studies should analyse how other tumor cell lines act following ^213^Bi-anti-EGFR-MAb treatment with regard to [^18^F]FDG-uptake and pyruvate conversion as well as *in vivo* studies as this data only represents *in vitro* observations and can only transferred to *in vivo* situations with precaution. The results of such studies might contribute to the future use these techniques in therapy monitoring of a variety of diseases. Moreover, the optimal time-point after ^213^Bi-anti-EGFR-MAb treatment for detection of ^213^Bi induced effects has to be ascertained. All in all, future studies hold promise to further underline the potential of hyperpolarized [1-^13^C]pyruvate in questions concerning treatment evaluation.

## Conclusions

Treatment with ^213^Bi-anti-EGFR-MAb resulted in an effective induction of cell death in EJ28Luc and LN18 cells. Lactate/pyruvate ratios of hyperpolarized [1-^13^C]pyruvate proved to detect early treatment response effects, which can be used to asses early treatment response. In combination with [^18^F]FDG-uptake valuable information about tumor metabolism can be gained, holding promise for future clinical applications in early therapy monitoring.

## Data Availability

Data generated or analysed during this study are included in this published article.
